# Nurses’ perception of safety barriers in nursing: content analysis

**Published:** 2019-08

**Authors:** Narjes Heshmatifar, Raha Saleh abadi, Mojgan Ansari, Fateme Borzoee

**Affiliations:** ^*a*^Sabzevar University of Medical Sciences, Sabzevar, Iran.; ^*b*^Department of Operating Room, School of Paramedics, Sabzevar University of Medical Sciences, Sabzevar, Iran.

## Abstract

**Background:**

Patient s safety is one of the important dimensions of care qualities. Care techniques, technology and humanities factors threaten patients’ safety. This study aimed to understand nurses’ perceptions of safety barriers in nursing profession.

**Methods:**

This qualitative study was carried out using semi-structured in-depth interviews with 12 nurses and two focused groups. Data were analyzed using Bernard s content analysis.

**Results:**

Having analyzed the extracted themes, three main themes including the individual features (the lack of specialized knowledge, the lack of awareness of law, responsibility, ability, job satisfaction and experience), management (work plan, educational program, salary and benefits, working conditions) and environment (inadequate equipment) were found.

**Conclusions:**

The results of the current study showed that personal characteristics, management plans and clinical environment have effects on safety. This study presents a plan for the classification of safety barriers that provides a framework for quantitative and qualitative research.

**Keywords:**

Safety, Nurses, Barriers, Content analysis

